# Supplementary material for: Wheat NAC transcription factor NAC5‐1 is a positive regulator of senescence
Source: Plant Direct. 2024 Jul 2;8(7):e620. doi: 10.1002/pld3.620 (PMC11217990; doi:10.1002/pld3.620)
Supplement: Supplementary file 4 — Figure S1. Crossing scheme for generation of NAC5‐1 TILLING mutant lines. A missense mutation in NAC5‐A1 (K2546) was crossed independently with missense mutations in NAC5‐B1 in (a) K2036 and (b) K3328. Two backcrosses to non‐mutagenized Kronos were carried out. Mutations in NAC5‐1 were tracked with KASP genotyping at each generation. BC2F3 lines were used for senescence phenotyping. Figure S2. Schematic of construct for NAC5‐A1 overexpression. The T‐DNA region of plasmid pMSH30 includes the nptII gene under an Sc4 promoter and NAC5‐A1 with a 5’ 3xFLAG tag under the rice Actin promoter. Figure S3. TraesCS4A02G219700 and its homoeologs are the orthologs of OsNAC5 and are expressed in senescence. (a) Alignment of OsNAC5, NAC5‐A1, NAC5‐B1, and NAC5‐D1 peptide sequences in Clustal Omega. Red marks sites with identity to OsNAC5 sequence. Conserved NAC subdomains of OsNAC5 are annotated from (Kikuchi et al., 2000). (b) Peptide sequences of the 15 top BLAST hits of OsNAC5 in Triticum aestivum and Oryza sativa ssp. japonica were aligned in Clustal Omega. A rooted tree was built with the Neighbor‐Joining method. The node containing OsNAC5 is highlighted. Numbers show support values from 100 bootstrap replicates. Scale bar shows number of substitutions per site. (c) Expression level of NAC5‐A1, NAC5‐B1, and NAC5‐D1 in transcripts per million (tpm) in flag leaves from 3DAA (days after anthesis) to 26DAA. Data from (Borrill et al., 2019). Figure S4. Additional traits in double mutants of NAC5‐1. (a) Main tiller number, (b) Grain number per tiller, (c) Average grain length (mm), (d) Average grain width (mm), (e) Average grain area (mm2), (f) Predicted grain protein content by NIR spectrometry, subset of plants with grain mass >15 g, dry basis (%), (g) Height of primary tiller (mm). (a‐g) ANOVA with post‐hoc Tukey test, formula ~ Row + Block + Genotype, letters show significance groups at p < .05. Data from two crosses were combined (n = 14–24). Figure S5. Selection of NAC5‐A1 tra [file PLD3-8-e620-s004.docx]

**Supplementary Figures and Tables for Evans *et al*. “Wheat NAC transcription factor *NAC5-1* is a positive regulator of senescence”.**


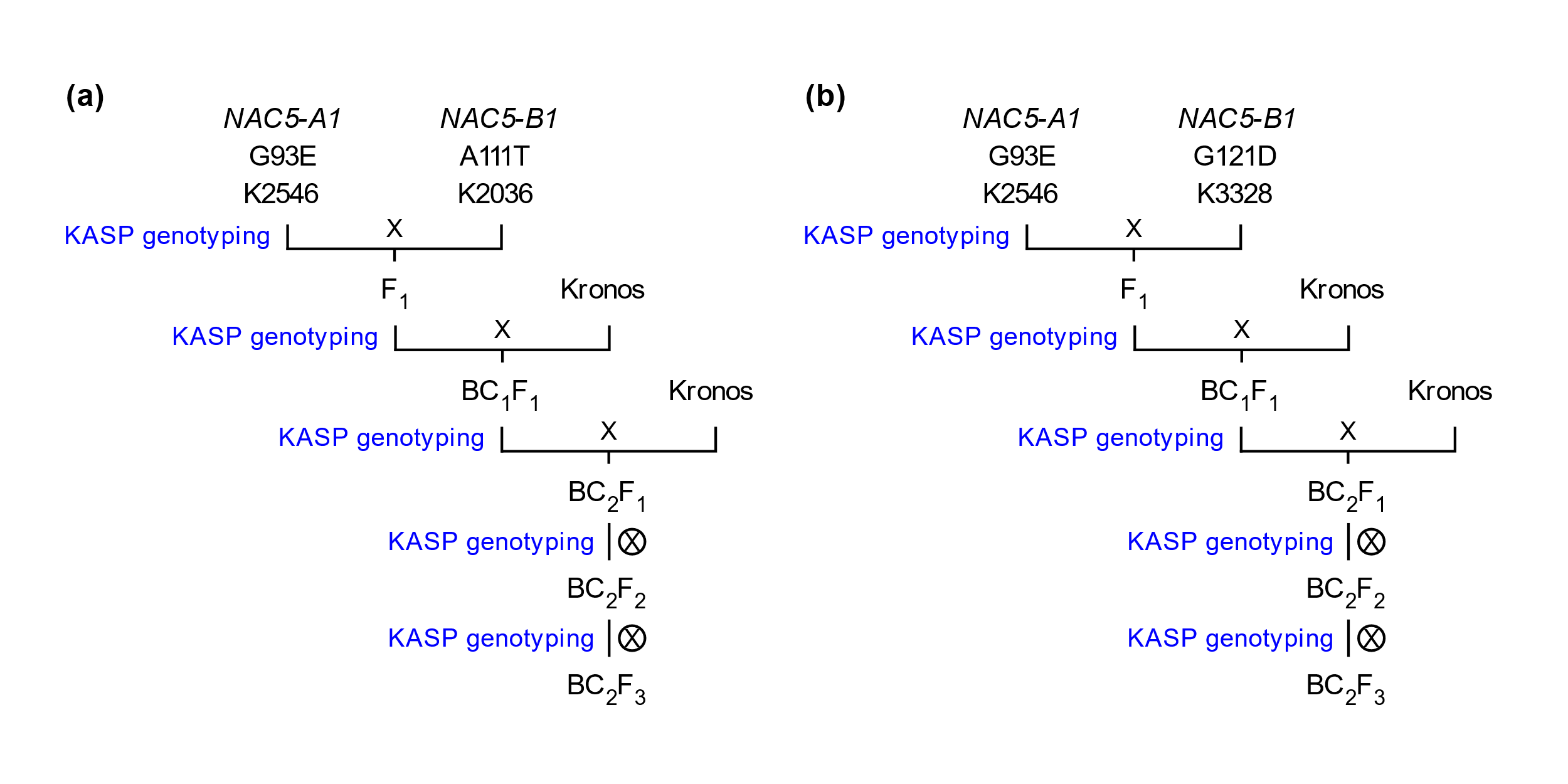
**Supplementary Figure 1.** Crossing scheme for generation of *NAC5-1* TILLING mutant lines. A missense mutation in *NAC5-A1* (K2546) was crossed independently with missense mutations in *NAC5-B1* in (a) K2036 and (b) K3328. Two backcrosses to non-mutagenized Kronos were carried out. Mutations in *NAC5-1* were tracked with KASP genotyping at each generation. BC_2_F_3_ lines were used for senescence phenotyping.

**
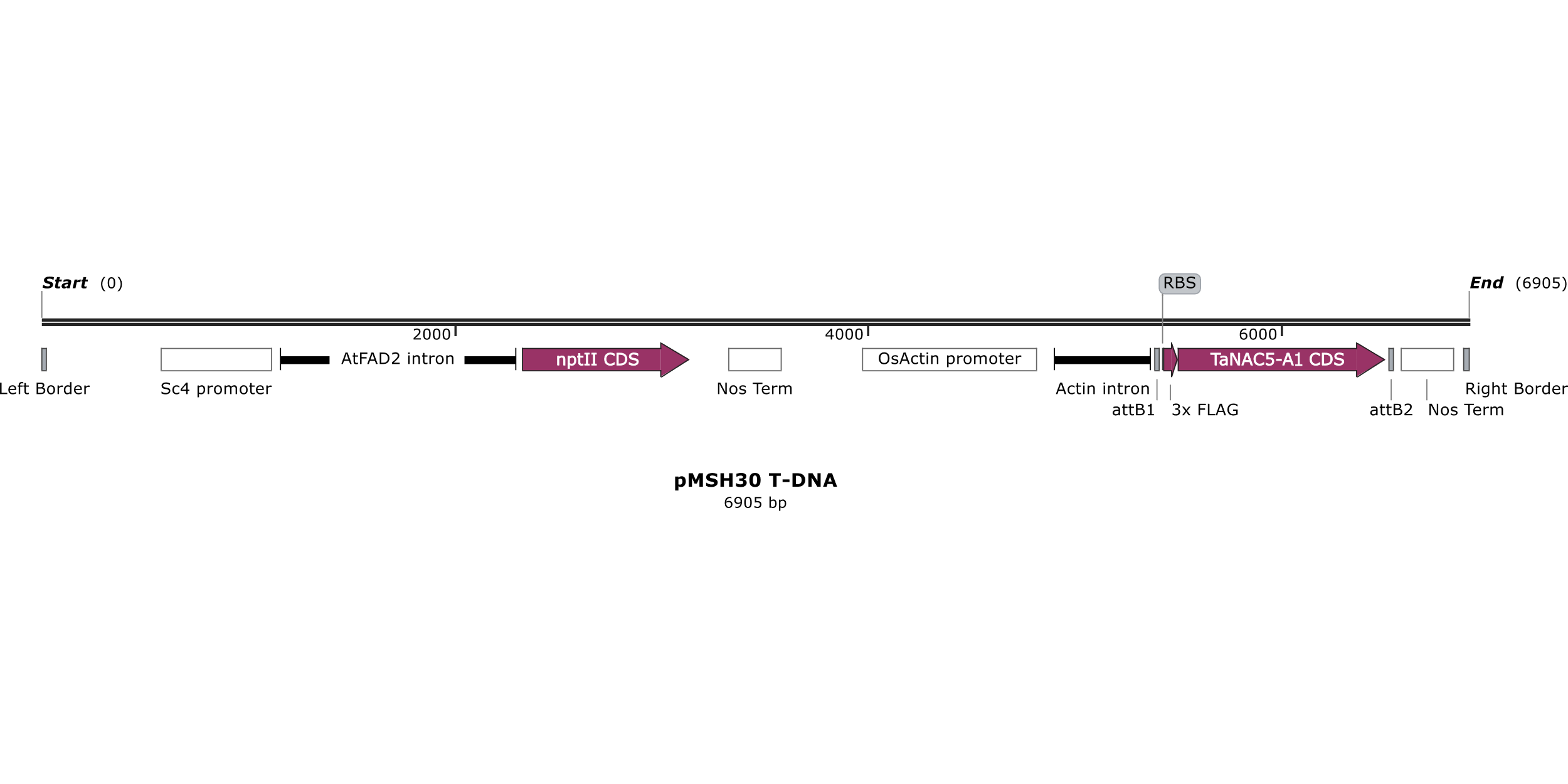
**

**Supplementary Figure 2.** Schematic of construct for *NAC5-A1* overexpression. The T-DNA region of plasmid pMSH30 includes the *nptII* gene under an Sc4 promoter and *NAC5-A1* with a 5’ 3xFLAG tag under the rice Actin promoter.

**
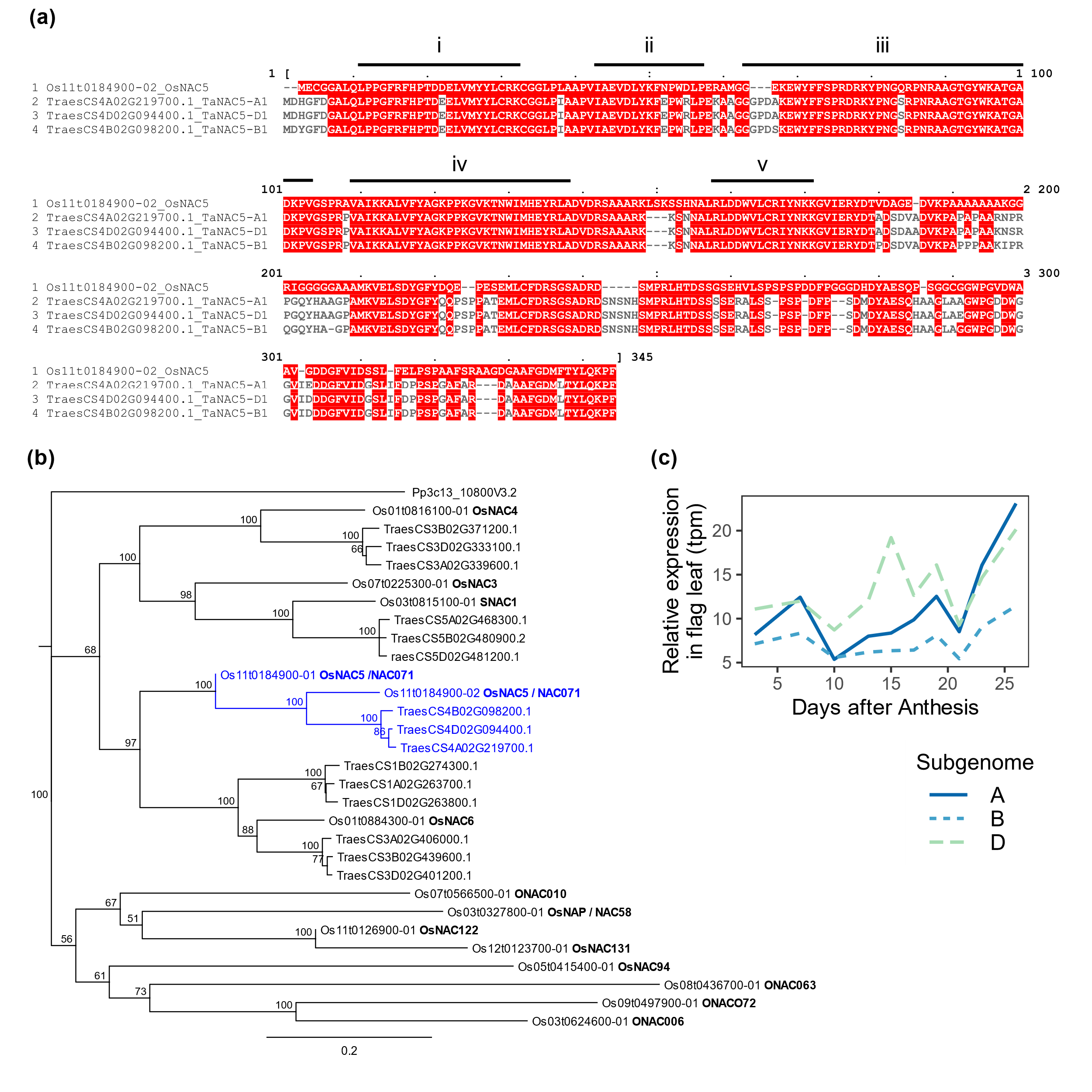
Supplementary Figure 3.** *TraesCS4A02G219700* and its homoeologs are the orthologs of *OsNAC5* and are expressed in senescence. (a) Alignment of *OsNAC5, NAC5-A1*, *NAC5-B1* and *NAC5-D1* peptide sequences in Clustal Omega. Red marks sites with identity to *OsNAC5* sequence. Conserved NAC subdomains of *OsNAC5* are annotated from (Kikuchi et al., 2000). (b) Peptide sequences of the 15 top BLAST hits of *OsNAC5* in Triticum aestivum and *Oryza sativa* ssp. *japonica* were aligned in Clustal Omega. A rooted tree was built with the Neighbour-Joining method. The node containing *OsNAC5* is highlighted. Numbers show support values from 100 bootstrap replicates. Scale bar shows number of substitutions per site. (c) Expression level of *NAC5-A1, NAC5-B1* and *NAC5-D1* in transcripts per million (tpm) in flag leaves from 3DAA (days after anthesis) to 26DAA. Data from (Borrill et al. 2019).


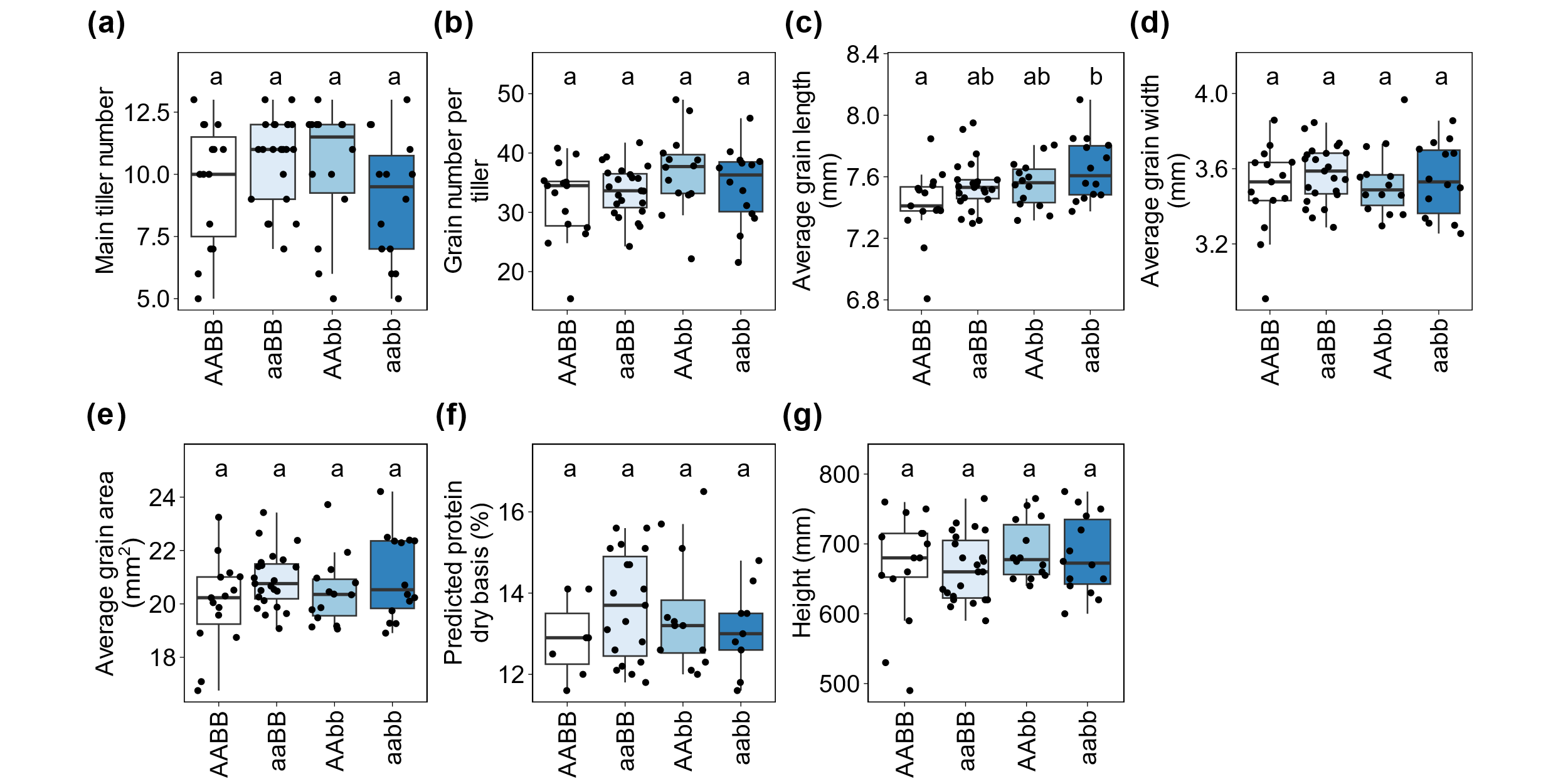
**Supplementary Figure 4.** Additional traits in double mutants of *NAC5-1*. (a) Main tiller number, (b) Grain number per tiller, (c) Average grain length (mm), (d) Average grain width (mm), (e) Average grain area (mm^2^), (f) Predicted grain protein content by NIR spectrometry, subset of plants with grain mass >15g, dry basis (%), (g) Height of primary tiller (mm). (a-g) ANOVA with post-hoc Tukey test, formula ~ Row + Block + Genotype, letters show significance groups at p<0.05. Data from two crosses were combined (n=14-24).

**
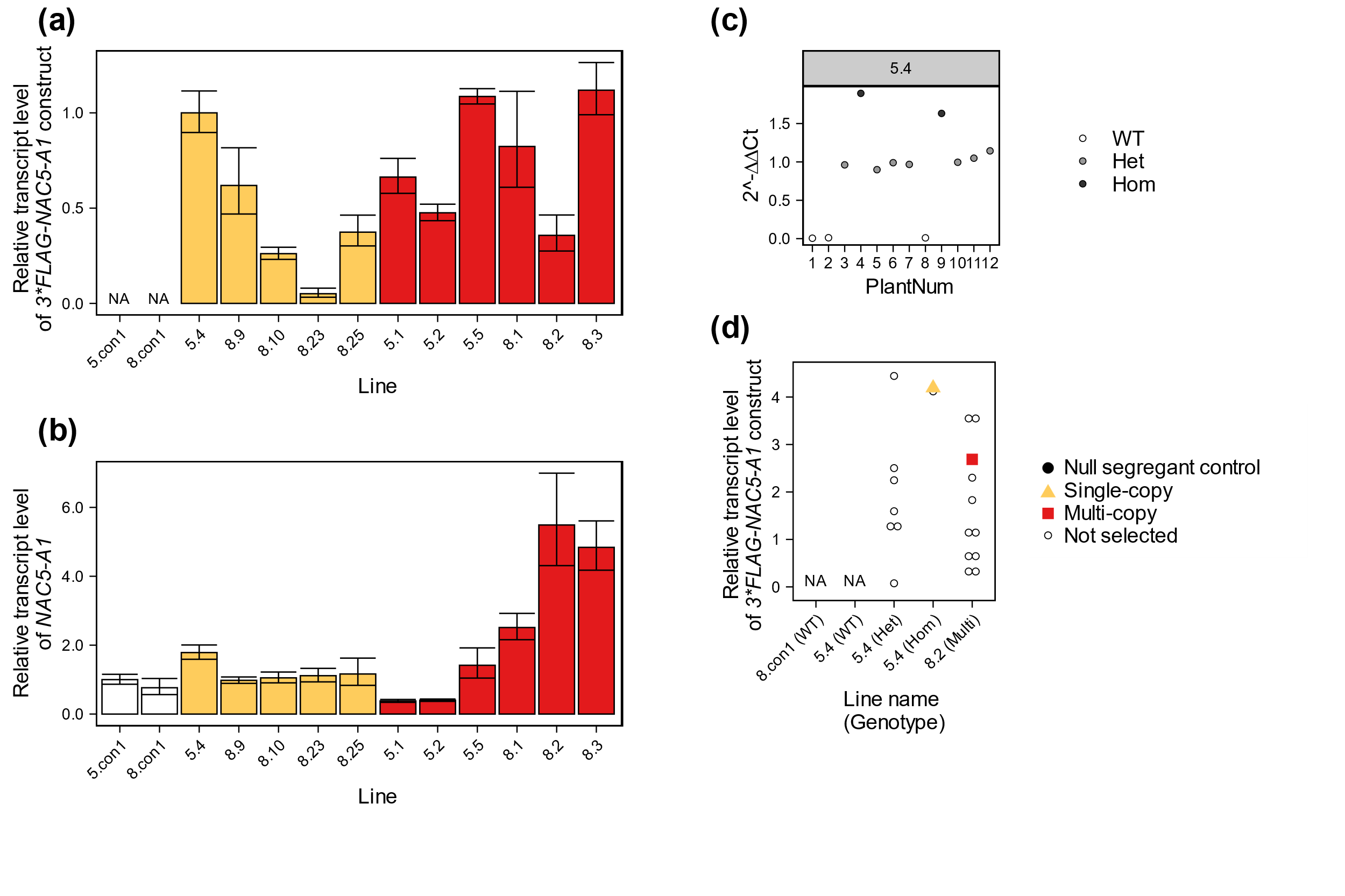
Supplementary Figure 5.** Selection of *NAC5-A1* transgenic lines. (a, b) Relative transcript level of pooled 3-week-old T_1_ leaf samples was analysed by qPCR using ΔΔCt method with Actin as reference gene. Bar plot and error bars show mean ±SD (n=3 technical replicates). “NA” marks samples not amplified. (a) Relative transcript level of 3*FLAG::*NAC5-A1* construct normalized against 5.4. (b) Relative transcript level of *NAC5-A1* normalized against 5.con1. (c) Construct genotypes of T_1_ plants in line 5.4 determined by copy number assay. Relative quantity of genomic DNA of marker gene *nptII* was analysed using Taqman probes and ΔΔCt method, normalised against single copy gene *GAMYB* and average of “Het” cluster. Colours show assigned genotypes. (d) Relative transcript level of individual 3-week-old T_1_ leaf samples was analysed using the Pfaffl method with primer efficiencies and Actin as reference gene, average of 3 technical replicates normalized against average of single copy lines. Non-transformed control (8.con1), single-copy line (5.4) categorised by construct genotype, and multi-copy line (8.2) are shown. Highlighted points mark T_1_ plants for which T_2_ progeny were used for phenotyping.


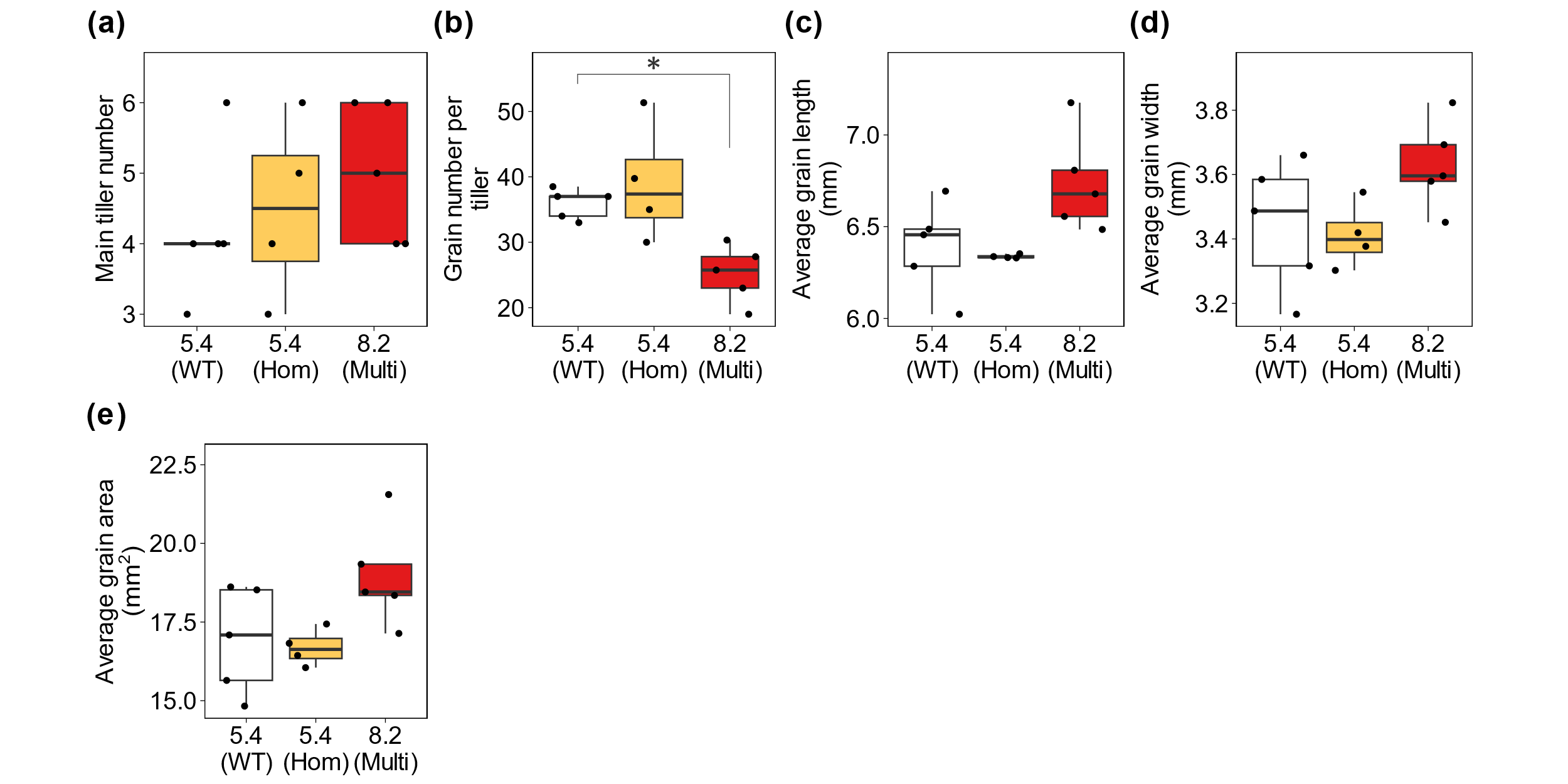
**Supplementary Figure 6.** Further traits in *NAC5-1* T_2_ transgenic lines. (a) Main tiller number, (b) Grain number per tiller, (c) Average grain length (mm), (d) Average grain width (mm), (e) Average grain area (mm^2^). (a-e) Pairwise comparisons by Wilcoxon test, ns = not significant; *=p<0.05; **=p<0.01 (n=4-5).

**Supplementary Table 1.** Primers used in this study. The third column corresponds to the Methods section in which these primers were used. Primers sourced from: [1] This study; [2] (Tenea et al., 2011); [3] (Milner et al., 2018); [4] Primers or modification of adapter sequences sourced from NEB.

| Primer name | Sequence | Experiment | Amplifies | Source |
| --- | --- | --- | --- | --- |
| CENAC5-1-F | GCTCAGGCTGGATGACT | qPCR | *NAC5-A1* | [1] |
| CENAC-7-R | GGCTTGACGTCGGCCA | qPCR | *NAC5-A1* | [1] |
| CENAC5-3-F | TGGATCATGCACGAGTACCG | qPCR | *NAC5-1 (all homoeologs)* | [1] |
| CENAC5-3-R | CACCCAGTCATCCAGCCTG | qPCR | *NAC5-1 (all homoeologs)* | [1] |
| CENAC5-5-F | AAGGACGATGACGATAAGGAC | qPCR | *3*FLAG-NAC5-A1* construct | [1] |
| CENAC5-5-R | GCAAAGGTAGTACATCACCAGC | qPCR | *3*FLAG-NAC5-A1* construct | [1] |
| PB441_Act2_F | CAAATCATGTTTGAGACCTTCAATG | qPCR | *ACT2* | [2] |
| PB441_Act2_R | ACCAGAATCCAACACGATACCTG | qPCR | *ACT2* | [2] |
| PB479_COM | tacttcttctcgccgcgC | KASP | *NAC5-A1* missense in Kronos2546 | [1] |
| PB479_MUT | GAAGGTGACCAAGTTCATGCTcGgtggccttccagtacT | KASP | *NAC5-A1* missense in Kronos2546 | [1] |
| PB479_WT | GAAGGTCGGAGTCAACGGATTcGgtggccttccagtacC | KASP | *NAC5-A1* missense in Kronos2546 | [1] |
| PB480_COM | gggtactggaaggccacG | KASP | *NAC5-B1* missense in Kronos2036 | [1] |
| PB480_MUT | GAAGGTGACCAAGTTCATGCTacgagAgccttcttgatggT | KASP | *NAC5-B1* missense in Kronos2036 | [1] |
| PB480_WT | GAAGGTCGGAGTCAACGGATTacgagAgccttcttgatggC | KASP | *NAC5-B1* missense in Kronos2036 | [1] |
| PB481_COM | gcaaggtggttaccctgagA | KASP | *NAC5-B1* missense in Kronos3328 | [1] |
| PB481_MUT | GAAGGTGACCAAGTTCATGCTtctcgtcttctacgccgA | KASP | *NAC5-B1* missense in Kronos3328 | [1] |
| PB481_WT | GAAGGTCGGAGTCAACGGATTtctcgtcttctacgccgG | KASP | *NAC5-B1* missense in Kronos3328 | [1] |
| GamyB1F | GATCCGAATAGCTGGCTCAAGTAT | Copy number | *GAMYB* | [3] |
| GamyB2R | GGAGACTGCAGGTAGGGATCAAC | Copy number | *GAMYB* | [3] |
| GamyB1P | [Joe]CGTGGCTCCTGCGATGCAGC[TAMRA] | Copy number | *GAMYB* | [3] |
| Npt2B2F | CTCCTGCCGAGAAAGTATCCA | Copy number | *nptII* | [3] |
| Npt2B4R | GCCGGATCAAGCGTATGC | Copy number | *nptII* | [3] |
| Npt2B2P | [FAM]TGGCTGATGCAATGCGGCG[TAMRA] | Copy number | *nptII* | [3] |
| M13F (-21) | TGTAAAACGACGGCCAGT | DAP-seq | universal vector sequencing | [4] |
| M13R | CAGGAAACAGCTATGAC | DAP-seq | universal vector sequencing | [4] |
| SM_P5_AMP | ACACTCTTTCCCTACACGACGCTCTTCCGATCT | DAP-seq | Illumina Truseq library adapters | [4] |
| SM_P7_AMP | GTGACTGGAGTTCAGACGTGTGCTCTTCCGATCT | DAP-seq | Illumina Truseq library adapters | [4] |
| TaNAC5-A-F | CCACCATGGACTACAAAGACCATGATGGAGAC  TATAAGGATCACGACATCGATTACAAGGACGAT  GACGATAAGGACCACGGCTTCGACG | Wheat transformation | *NAC5-A1,* adding RBS and 3*FLAG | [1] |
| TaNAC5-A-R2 | TCAGAACGGCTTCTGCAGG | Wheat transformation | *NAC5-A1,* adding RBS and 3*FLAG | [1] |

**Supplementary Table 2.** Summary of copy number analysis of *NAC5-A1* T_0_ transgenic plants. Copy number analysis was carried out for marker gene *nptII.*

| Copy number | Number of independently transformed plants |
| --- | --- |
| 1 | 5 |
| 2 | 4 |
| 3 | 1 |
| 4+ | 25 |
| Ambiguous | 8 |
| Non-transformed control | 3 |

**Supplementary Table 3.** Plant growth conditions. Metadata, environmental conditions and experimental design variables (where applicable) are supplied. Watering in all experiments was delivered by automatic flood benching.

| Sowing date | Harvest date | Plant material | Location | Light | Temperature | Pot size | Compost | Other environmental conditions | Experimental layout | Replicates per genotype | Timing of SPAD reading |
| --- | --- | --- | --- | --- | --- | --- | --- | --- | --- | --- | --- |
| 16/11/2020 | 05/03/2021 | *NAC5-A1* overexpression T_1_ | Glasshouse | 16L:8D Supplementary | 20˚C day/ 18˚C night minimum | 11cm | 3:1 compost:  perlite | Standard practice fertiliser and fungicide treatments | NA | NA | NA |
|  |  |  |  |  |  |  |  |  |  |  |  |
| 23/12/2021 | 20/04/2022 | *NAC5-A1* overexpression T_2_ | Controlled Environment Chamber | 16L:8D LED 300µMol | 20˚C day/ 15˚C night | 9cm | John Innes Cereal Mix | 70% humidity, 3 fungicide treatments | RCBD | 10 | At Heading, Weekly 6-8DAH to SPAD<10 |
|  |  |  |  |  |  |  |  |  |  |  |  |
| 01/12/2021 | 19/04/2022 | *NAC5-1* Kronos TILLING | Glasshouse | 16L:8D Supplementary | 20˚C day/ 18˚C night minimum | 1L | John Innes Cereal Mix | Standard practice fertiliser and fungicide treatments | RCBD | 12 | At Heading, Weekly 6-8DAH to SPAD<10 |

**Supplementary Table 4.** Gene IDs used in this study. Gene IDs refer to [1] *Triticum aestivum* assembly IWGSC RefSeq v1.1, [2] NCBI Genbank, or [3] *Oryza sativa* assembly IRGSP-1.0. Gene names coined by [4] This study, [5] (Uauy et al., 2006), [6] (Kikuchi et al., 2000).

| Gene ID | Gene name in this study | Gene ID source | Gene name source |
| --- | --- | --- | --- |
| TraesCS4A02G219700 | *NAC5-A1* | [1] | [4] |
| TraesCS4B02G098200 | *NAC5-B1* | [1] | [4] |
| TraesCS4D02G094400 | *NAC5-D1* | [1] | [4] |
| TraesCS6A02G108300 | *NAM-A1* | [1] | [5] |
| DQ869673 | *NAM-B1* | [2] | [5] |
| TraesCS6D02G096300 | *NAM-D1* | [1] | [5] |
| TraesCS2A02G201800 | *NAM-A2* | [1] | [5] |
| TraesCS2B02G228900 | *NAM-B2* | [1] | [5] |
| TraesCS2D02G214100 | *NAM-D2* | [1] | [5] |
| Os11g0184900 | *OsNAC5* | [3] | [6] |
