# Supplementary material for: Wheat NAC transcription factor NAC5‐1 is a positive regulator of senescence
Source: Plant Direct. 2024 Jul 2;8(7):e620. doi: 10.1002/pld3.620 (PMC11217990; doi:10.1002/pld3.620)
Supplement: Supplementary file 1 — Dataset S1. Peer Review [file PLD3-8-e620-s002.pdf]

Plant Direct
Open Access

Wheat NAC transcription factor *NAC5-1* is a positive regulator of senescence

Catherine Evans[1, 2], Sophie Louise Mogg[3], Charline Soraru[4], Emma Wallington[4], Juliet Coates[2], Philippa Borrill[1†]

Decision Letter Round 1:

May 5, 2024

Dr. Philippa Borrill
John Innes Centre
Crop Genetics
Norwich Research Park
Norwich NR4 7UH
United Kingdom

RE: Wheat NAC transcription factor NAC5-1 is a positive regulator of senescence

Dear Dr. Borrill:

Thank you for submitting to Plant Direct. All required reviews have been returned and we have now finished our evaluation of your manuscript. In light of the reviewers' and editor's comments, further revisions are needed before the paper can be accepted for publication in Plant Direct.

Please view the editors' and reviewers' comments below and use their suggestions as a guide while you work on your revision.

When uploading the revised version of this article, please be sure to include the following:

-A word document that contains your response to the reviewers. You should respond to each reviewer comment and note the changes made to the manuscript. If you do not agree with a reviewer's comment and choose not to make a suggested revision, please explain why. Please try to provide as complete an answer as possible to each reviewer's criticisms.
-A tracked changes document with each change highlighted

- A clean version of the latest version of the manuscript

First, let me apologize for the slow response. I had a difficult time finding reviewers for the manuscript, a worrisome current trend for all manuscripts. I secured one reviewer, and I also reviewed the manuscript. I found the manuscript compelling and well-written. The discussion does a good job at highlighting the limitations of the work and the conclusions. Overall, I think it is almost acceptable for publication, although I have a couple of small comments below, and the reviewer also ask for some details. It is important to note that Plant Direct policy is not to request new experiments unless they current experiments are not technically sound, and this is not the case for your manuscript. I would suggest that you evaluate the comments from myself and the other reviewer and either make the minor changes requested or provide a rebuttal.
My specific comments: In your analysis of leaf chlorophyll you used SPAD, and the results are expressed in SPAD units. Please provide and explanation of what is measured by the devise, and what the value means. Why do you choose a "below 10" threshold? Is this a % value? Also, in Section 3.1 the manuscript describes the orthologs of NAC5-1 in hexaploidy wheat, but section 3.2 uses a tetraploid wheat for functional analysis. Please make it clear that the tetraploid has only the A and B genomes and so only the A and B orthologs are present (there is no mistake in your manuscript, but I think that the clarification will make it easier for readers to understand the results).

To upload your revision, please click the link below.
https://plantdirect.msubmit.net/cgi-bin/main.plex?el=A2Lr7gY6A5IRc1I7A9ftd9hvHPvnDxpntqhZCKhq54gZ

In order to provide as timely a service as possible, we ask that your revision is resubmitted within three months after receipt of this request. If an extension is needed, please send a request, along with a brief explanation, to the editorial office at plantdirect@wiley.com .

Please note that, in addition to publishing reviewer comments, the author's responses to review comments will also be published alongside the final version of the paper. If you would not like the author's responses to be published, please contact the editorial office at plantdirect@wiley.com .

Thank you very much for giving us an opportunity to review your work. I look forward to receiving the next version.

Sincerely,

Gustavo MacIntosh

Gustavo MacIntosh

Editor, Plant Direct

------------------------------------------------------------------------------ Editor comments:
First, let me apologize for the slow response. I had a difficult time finding reviewers for the manuscript, a worrisome current trend for all manuscripts. I secured one reviewer, and I also reviewed the manuscript. I found the manuscript compelling and well-written. The discussion does a good job at highlighting the limitations of the work and the conclusions. Overall, I think it is almost acceptable for publication, although I have a couple of small comments below, and the reviewer also ask for some details. It is important to note that Plant Direct policy is not to request new experiments unless they current experiments are not technically sound, and this is not the case for your manuscript. I would suggest that you evaluate the comments from myself and the other reviewer and either make the minor changes requested or provide a rebuttal.
My specific comments: In your analysis of leaf chlorophyll you used SPAD, and the results are expressed in SPAD units. Please provide and explanation of what is measured by the devise, and what the value means. Why do you choose a "below 10" threshold? Is this a % value? Also, in Section 3.1 the manuscript describes the orthologs of NAC5-1 in hexaploidy wheat, but section 3.2 uses a tetraploid wheat for functional analysis. Please make it clear that the tetraploid has only the A and B genomes and so only the A and B orthologs are present (there is no mistake in your manuscript, but I think that the clarification will make it easier for readers to understand the results).

------------------------------------------------------------------------------ Reviewer comments:
Reviewer #1:

Dear authors:

In this manuscript (ID: 2024-01442), you identified missense mutations in NAC5-A1 and NAC5-B1 from a TILLING mutant population, and overexpressed NAC5-A1 in wheat as well. Mutation of NAC5-1 resulted in late leaf senescence and overexpression of NAC5-1 led to early leaf senescence. DNA affinity purification assays indicated that known senescence associated genes might be the downstream targets of NAC5-1.
It is a straight-forward study and I believe the manuscript is prepared well and main results support the initial design. The findings are meaningful and interesting for our understanding toward to the function of NAC5-1 (an ortholog of OsNAC5) in wheat. And it also could be used to improve wheat molecular breeding. Thus, from a content point of view, I like the manuscript.

However, following comments are encouraged to consider or address when improving the manuscript:
(1) There are no any pictures showing whole wheat plants or leaves in the manuscript.
(2) It would be better to provide the data of molecular maker genes of leaf senescence besides the data of leaf chlorophyll content (SPAD), in Figure 1, 2
(3) To confirm the validation of DAP-seq, at least a couple of identified putative downstream targets of NAC5-1 should be verified through biochemical assays (EMSA, BiFC, ChIP, etc).

Decision Letter Round 2:

June 10, 2024
Dr. Philippa Borrill
John Innes Centre
Crop Genetics
Norwich Research Park
Norwich NR4 7UH
United Kingdom

MSID: 2024-01442R1
MS TITLE: Wheat NAC transcription factor NAC5-1 is a positive regulator of senescence

Dear Dr. Philippa Borrill:

I am pleased to inform you that your manuscript "Wheat NAC transcription factor NAC5-1 is a positive regulator of senescence" has been accepted for publication in Plant Direct.

Your article will appear online in the next available issue of Plant Direct. To ensure your article gets published as quickly as possible, please pay attention to the steps detailed below. We have found that most of the delays happen at this stage, especially at the payment stage, so please respond as quickly as possible when prompted.

License Agreement: Once your article has been accepted it will move to Production and undergo admin and file checking - you may receive an email with any queries we have at this stage. When all required items are received by the publisher and queries resolved, the corresponding author will receive an email from Wiley's Author Services system which will ask them to log on at https://authorservices.wiley.com/bauthor and will present them with the appropriate license for completion. Your article cannot be published until both the signed license agreement and payment of the article fee have been received.

Payment of the Open Access Article Publication Fee: All articles published in Plant Direct are fully open access: immediately and freely available to read, download and share. Plant Direct charges a publication fee to cover the publication costs. The corresponding author for this manuscript should have already received a quote from the payments team (cs-openaccess@wiley.com) with the estimated article publication fee; please email cs-openaccess@wiley.com if this has not been received. The corresponding author should log on to the Wiley Author Services site, where the publication fee can be paid by credit card or an invoice. Pro Forma can also be requested. Payment of the publication charge must be received before the article will be published online.

Proofs: You will have the opportunity to look over your paper once more when you receive the author proofs for your article. The proofs will be with you in approximately two weeks. Please note that, in addition to publishing reviewer comments, the author's responses to review comments will also be published alongside the final version of the paper. If you would not like the author's responses to be published, please contact the editorial office at plantdirect@wiley.com .

Promotion of your article: You can help your research get the attention it deserves! Check out Wiley's free Promotion Guide for best-practice recommendations for promoting your work at www.wileyauthors.com/eeo/guide. And learn more about Wiley Editing Services which offers professional video, design, and writing services to create shareable video abstracts, infographics, conference posters, lay summaries, and research news stories for your research at www.wileyauthors.com/eeo/promotion.

Thank you again for your contribution to Plant Direct. If you have any questions, feel free to contact the editorial office at plantdirect@wiley.com .

Sincerely,

Gustavo MacIntosh

Gustavo MacIntosh

Editor, Plant Direct

------------------------------------------------------------------------------ Editor comments

------------------------------------------------------------------------------ Reviewer comments:

Author's Response:

Editor comments: First, let me apologize for the slow response. I had a difficult time finding reviewers for the manuscript, a worrisome current trend for all manuscripts. I secured one reviewer, and I also reviewed the manuscript. I found the manuscript compelling and well-written. The discussion does a good job at highlighting the limitations of the work and the conclusions. Overall, I think it is almost acceptable for publication, although I have a couple of small comments below, and the reviewer also ask for some details. It is important to note that Plant Direct policy is not to request new experiments unless they current experiments are not technically sound, and this is not the case for your manuscript. I would suggest that you evaluate the comments from myself and the other reviewer and either make the minor changes requested or provide a rebuttal. Thank you for your efforts to secure reviewers and for reviewing the manuscript yourself. We address each of the comments in turn below. My specific comments: In your analysis of leaf chlorophyll you used SPAD, and the results are expressed in SPAD units. Please provide and explanation of what is measured by the devise, and what the value means. Why do you choose a "below 10" threshold? Is this a % value? In

Methods Section 2.9, we have added the following explanation of SPAD values. “The SPAD spectrometer measures the optical density difference of red (650nm) and near-infrared (940nm) wavelengths. In wheat, SPAD values show a strong correlation (r2 = 0.8-0.9) with leaf chlorophyll content estimated from chemical extraction” and that “A threshold of 10 was used as SPAD values below 10 were found to be less reproducible.” It was also added where SPAD is first introduced in the Results (Section 3.2) that “A SPAD spectrometer was used as a non-destructive indicator of flag leaf chlorophyll contents, expressed in SPAD units.” Also, in Section 3.1 the manuscript describes the orthologs of NAC5-1 in hexaploidy wheat, but section 3.2 uses a tetraploid wheat for functional analysis. Please make it clear that the tetraploid has only the A and B genomes and so only the A and B orthologs are present (there is no mistake in your manuscript, but I think that the clarification will make it easier for readers to understand the results). We have added a comment in Section 3.2 that “Tetraploid wheat was selected to accelerate the crossing program as the tetraploid wheat genome has two subgenomes, A and B, hence contains only two homoeologs of NAC5-1, NAC5-A1 and NAC5-B1.” In Section 3.1 we clarified that the description and expression data refer to hexaploid wheat. Reviewer comments: Reviewer #1: Dear authors: In this manuscript (ID: 2024-01442), you identified missense mutations in NAC5-A1 and NAC5-B1 from a TILLING mutant population, and overexpressed NAC5-A1 in wheat as well. Mutation of NAC5- 1 resulted in late leaf senescence and overexpression of NAC5-1 led to early leaf senescence. DNA affinity purification assays indicated that known senescence associated genes might be the downstream targets of NAC5-1. It is a straight-forward study and I believe the manuscript is prepared well and main results support the initial design. The findings are meaningful and interesting for our understanding toward to the function of NAC5-1 (an ortholog of OsNAC5) in wheat. And it also could be used to improve wheat molecular breeding. Thus, from a content point of view, I like the manuscript. However, following comments are encouraged to consider or address when improving the manuscript: (1) There are no any pictures showing whole wheat plants or leaves in the manuscript. In the experiments with NAC5-1 overexpression lines and BC2 TILLING lines presented, the differences in senescence between genotypes were measurable but subtle, and the variability in heading time limited opportunities to collect photographs. However, an illustrative photograph from a NAC5-1 BC1 TILLING line has been added as Figure 1b, and the figure legend modified. Results Section 3.2 now states “In preliminary trials at the BC1 generation, delayed leaf senescence was observed in NAC5-1 double mutant lines compared to controls (Figure 1b).” (2) It would be better to provide the data of molecular maker genes of leaf senescence besides the data of leaf chlorophyll content (SPAD), in Figure 1, 2 Thank you for this comment. The primary objective of this paper was to investigate the impact on plant phenotypes (such as flag leaf chlorophyll content, peduncle senescence and grain traits) in NAC5-1 overexpression and mutant lines. Thus, qPCR data on the expression of

senescenceassociated marker genes is outside the scope of this paper. (3) To confirm the validation of DAP-seq, at least a couple of identified putative downstream targets of NAC5-1 should be verified through biochemical assays (EMSA, BiFC, ChIP, etc). As described in Section 3.5, we showed some corroboration of the DAP-seq in this study by comparison with previously published datasets predicting NAC5-1 targets (a GENIE3 network and an RNA-seq experiment). The discussion (section 4.3) highlighted putative target genes with evidence from more than one homoeolog and/or more than one method. Future work on the regulatory mechanism of NAC5-1 will include biochemical assays to validate downstream target genes, but we consider this would be more suited to a subsequent paper. We have acknowledged value of future biochemical validation at the end of Section 4.3 as follows: “In summary, some of the putative target genes of NAC5-1 identified from the DAP-seq from this study together with independent datasets may be connected with senescence and nitrogen remobilisation. Biochemical validation of these putative downstream targets, for example using electrophoretic mobility shift assays, chromatin immunoprecipitation or luciferase assays would be valuable future work to clarify the mechanism of senescence regulation by NAC5-1.”
