# Supplementary material for: Wheat NAC transcription factor NAC5‐1 is a positive regulator of senescence
Source: Plant Direct. 2024 Jul 2;8(7):e620. doi: 10.1002/pld3.620 (PMC11217990; doi:10.1002/pld3.620)
Supplement: Supplementary file 3 — Dataset S3. Supporting Information [file PLD3-8-e620-s003.pdf]

Editor comments:
First, let me apologize for the slow response. I had a difficult time finding reviewers for the manuscript, a worrisome current trend for all manuscripts. I secured one reviewer, and I also reviewed the manuscript. I found the manuscript compelling and well-written. The discussion does a good job at highlighting the limitations of the work and the conclusions. Overall, I think it is almost acceptable for publication, although I have a couple of small comments below, and the reviewer also ask for some details. It is important to note that Plant Direct policy is not to request new experiments unless they current experiments are not technically sound, and this is not the case for your manuscript. I would suggest that you evaluate the comments from myself and the other reviewer and either make the minor changes requested or provide a rebuttal.

Thank you for your efforts to secure reviewers and for reviewing the manuscript yourself. We address each of the comments in turn below.

My specific comments:

In your analysis of leaf chlorophyll you used SPAD, and the results are expressed in SPAD units. Please provide and explanation of what is measured by the devise, and what the value means. Why do you choose a "below 10" threshold? Is this a % value?
In Methods Section 2.9, we have added the following explanation of SPAD values. “The SPAD spectrometer measures the optical density difference of red (650nm) and near-infrared (940nm) wavelengths. In wheat, SPAD values show a strong correlation (r^2 = 0.8-0.9) with leaf chlorophyll content estimated from chemical extraction” and that “A threshold of 10 was used as SPAD values below 10 were found to be less reproducible.”

It was also added where SPAD is first introduced in the Results (Section 3.2) that “A SPAD spectrometer was used as a non-destructive indicator of flag leaf chlorophyll contents, expressed in SPAD units.”

Also, in Section 3.1 the manuscript describes the orthologs of NAC5-1 in hexaploidy wheat, but section 3.2 uses a tetraploid wheat for functional analysis. Please make it clear that the tetraploid has only the A and B genomes and so only the A and B orthologs are present (there is no mistake in your manuscript, but I think that the clarification will make it easier for readers to understand the results).
We have added a comment in Section 3.2 that “Tetraploid wheat was selected to accelerate the crossing program as the tetraploid wheat genome has two subgenomes, A and B, hence contains only two homoeologs of *NAC5-1*, *NAC5-A1* and *NAC5-B1*.”

In Section 3.1 we clarified that the description and expression data refer to hexaploid wheat.

Reviewer comments:
Reviewer #1:

Dear authors:
In this manuscript (ID: 2024-01442), you identified missense mutations in NAC5-A1 and NAC5-B1 from a TILLING mutant population, and overexpressed NAC5-A1 in wheat as well. Mutation of NAC5-1 resulted in late leaf senescence and overexpression of NAC5-1 led to early leaf senescence. DNA affinity purification assays indicated that known senescence associated genes might be the downstream targets of NAC5-1.
It is a straight-forward study and I believe the manuscript is prepared well and main results support the initial design. The findings are meaningful and interesting for our understanding toward to the

function of NAC5-1 (an ortholog of OsNAC5) in wheat. And it also could be used to improve wheat molecular breeding. Thus, from a content point of view, I like the manuscript.

However, following comments are encouraged to consider or address when improving the manuscript:
(1) There are no any pictures showing whole wheat plants or leaves in the manuscript.
In the experiments with *NAC5-1* overexpression lines and BC_2 TILLING lines presented, the differences in senescence between genotypes were measurable but subtle, and the variability in heading time limited opportunities to collect photographs. However, an illustrative photograph from a *NAC5-1* BC_1 TILLING line has been added as Figure 1b, and the figure legend modified.

Results Section 3.2 now states "In preliminary trials at the BC_1 generation, delayed leaf senescence was observed in *NAC5-1* double mutant lines compared to controls (Figure 1b)."

(2) It would be better to provide the data of molecular maker genes of leaf senescence besides the data of leaf chlorophyll content (SPAD), in Figure 1, 2
Thank you for this comment. The primary objective of this paper was to investigate the impact on plant phenotypes (such as flag leaf chlorophyll content, peduncle senescence and grain traits) in *NAC5-1* overexpression and mutant lines. Thus, qPCR data on the expression of senescence-associated marker genes is outside the scope of this paper.

(3) To confirm the validation of DAP-seq, at least a couple of identified putative downstream targets of NAC5-1 should be verified through biochemical assays (EMSA, BiFC, ChIP, etc).
As described in Section 3.5, we showed some corroboration of the DAP-seq in this study by comparison with previously published datasets predicting *NAC5-1* targets (a GENIE3 network and an RNA-seq experiment). The discussion (section 4.3) highlighted putative target genes with evidence from more than one homoeolog and/or more than one method.

Future work on the regulatory mechanism of *NAC5-1* will include biochemical assays to validate downstream target genes, but we consider this would be more suited to a subsequent paper.

We have acknowledged value of future biochemical validation at the end of Section 4.3 as follows: "In summary, some of the putative target genes of *NAC5-1* identified from the DAP-seq from this study together with independent datasets may be connected with senescence and nitrogen remobilisation. Biochemical validation of these putative downstream targets, for example using electrophoretic mobility shift assays, chromatin immunoprecipitation or luciferase assays would be valuable future work to clarify the mechanism of senescence regulation by *NAC5-1*."
